# RETRACTION: Impact of Negative Pressure Wound Treatment on Incidence of Surgical Site Infection in Varied Orthopedic Surgeries: A Systematic Review and Meta‐Analysis

**DOI:** 10.1111/iwj.70561

**Published:** 2025-04-18

## Abstract

**RETRACTION:** YuanS. , ZhangT. , ZhangD. , HeQ. , DuM. , and ZengF. , “Impact of Negative Pressure Wound Treatment on Incidence of Surgical Site Infection in Varied Orthopedic Surgeries: A Systematic Review and Meta‐Analysis,” International Wound Journal 20, no. 6 (2023): 2334–2345, 10.1111/iwj.14043.36524330 PMC10333009

The above article, published online on 16 December 2022, in Wiley Online Library (http://onlinelibrary.wiley.com/), has been retracted by agreement between the journal Editor in Chief, Professor Keith Harding; and John Wiley & Sons Ltd. Following an investigation by the publisher, all parties have concluded that this article was accepted solely on the basis of a compromised peer review process. The editors have therefore decided to retract the article. The authors did not indicate their agreement with the retraction.

**RETRACTION:** S. Yuan , T. Zhang , D. Zhang , Q. He , M. Du , and F. Zeng , “Impact of Negative Pressure Wound Treatment on Incidence of Surgical Site Infection in Varied Orthopedic Surgeries: A Systematic Review and Meta‐Analysis,” International Wound Journal 20, no. 6 (2023): 2334–2345, 10.1111/iwj.14043.36524330 PMC10333009

The above article, published online on 16 December 2022, in Wiley Online Library (http://onlinelibrary.wiley.com/), has been retracted by agreement between the journal Editor in Chief, Professor Keith Harding; and John Wiley & Sons Ltd. Following an investigation by the publisher, all parties have concluded that this article was accepted solely on the basis of a compromised peer review process. The editors have therefore decided to retract the article. The authors did not indicate their agreement with the retraction.

